# Experimental Assessment of the Water Quality Influence on the Phosphorus Uptake of an Invasive Aquatic Plant: Biological Responses throughout Its Phenological Stage

**DOI:** 10.1371/journal.pone.0118844

**Published:** 2015-03-18

**Authors:** Virginie Baldy, Gabrielle Thiebaut, Catherine Fernandez, Marketa Sagova-Mareckova, Nathalie Korboulewsky, Yogan Monnier, Thierry Perez, Michele Tremolieres

**Affiliations:** 1 Laboratoire d’Hydrologie et de Géochimie de Strasbourg (LHyGeS UMR 7517 UdS / CNRS), Institut de Botanique, 28 rue Goethe, F-67083, Strasbourg Cedex, France; 2 Aix Marseille Université, CNRS, IRD, Avignon Université, Institut Méditerranéen de Biodiversité et d’Ecologie IMBE UMR 7263, CS 80249, 13331, Marseille, Cedex 03, France; 3 Laboratoire Ecosystèmes-Biodiversité-Evolution-ECOBIO-UMR CNRS 6553, 263 av. Général Leclerc, F-35042, Rennes Cedex, France; 4 Epidemiology and ecology of microorganisms, Crop Research Institute, Prague, Czech Republic; 5 Irstea, UR EFNO, centre de Nogent-sur-Vernisson, F-45290, Nogent-sur-Vernisson, France; 6 INRA, UMR AMAP, Montpellier, F-34000, France; Texas A&M University at Galveston, UNITED STATES

## Abstract

Understanding how an invasive plant can colonize a large range of environments is still a great challenge in freshwater ecology. For the first time, we assessed the relative importance of four factors on the phosphorus uptake and growth of an invasive macrophyte *Elodea nuttallii* (Planch.) St. John. This study provided data on its phenotypic plasticity, which is frequently suggested as an important mechanism but remains poorly investigated. The phosphorus uptake of two *Elodea nuttallii* subpopulations was experimentally studied under contrasting environmental conditions. Plants were sampled in the Rhine floodplain and in the Northern Vosges mountains, and then maintained in aquaria in hard (Rhine) or soft (Vosges) water. Under these conditions, we tested the influence of two trophic states (eutrophic state, 100 μg.l^−1^ P-PO_4_^3−^ and hypertrophic state, 300 μg.l^−1^ P-PO_4_^3−^) on the P metabolism of plant subpopulations collected at three seasons (winter, spring and summer). *Elodea nuttallii* was able to absorb high levels of phosphorus through its shoots and enhance its phosphorus uptake, continually, after an increase of the resource availability (hypertrophic > eutrophic). The lowest efficiency in nutrient use was observed in winter, whereas the highest was recorded in spring, what revealed thus a storage strategy which can be beneficial to new shoots. This experiment provided evidence that generally, the water trophic state is the main factor governing P uptake, and the mineral status (softwater > hardwater) of the stream water is the second main factor. The phenological stage appeared to be a confounding factor to P level in water. Nonetheless, phenology played a role in P turnover in the plant. Finally, phenotypic plasticity allows both subpopulations to adapt to a changing environment.

## Introduction

A general theory of community susceptibility to biological invasions has not been clearly spelled out but recent studies confirmed the key role of phosphorus (P) availability in controlling development and abundance of invasive macrophytes in aquatic systems [1–3]. Nutrient-use efficiencies depend both on the plant P metabolism and on its ability to assimilate this nutrient within its vegetative structures [2]. A high interspecific variation in P storage ability within aquatic plants has already been observed [2, 4, 5], indeed, the “luxury” uptake may benefit the particular plant species if nutrient concentrations in the environment diminish [3, 6, 7]. Consequently, plants with high P storage capacities should be able to live under a wide range of ecological conditions [3]. This could explain effective competition strategies of aquatic [8] or terrestrial plants [9].

*Elodea nuttallii* (Planch.) St. John, an invasive plant introduced in European freshwaters, shows high capacity for storing P and mobilising this nutrient accumulated in plant tissue for growth, while displaying a very intensive growth rate and short doubling-times [10, 11]. Moreover, the invasiveness of *E*. *nuttallii* increases in eutrophic waters [3], with eutrophication encouraging a continued spreading [12]. Even though the relative importance of both compartments, water and sediment, appeared to have major importance in P uptake by macrophytes, other factors such as flow velocity, light, temperature, chemistry such as mineralization, season and population could act as key players in plant growth and physiology in some situations [13–15]. For example, Thiébaut [3] observed seasonal variability in P uptake by macrophytes, depending on phenology, morphology and the fluctuating resource availability in the sites. P-storage observed on an annual scale showed different patterns between species [4, 5]: *E*. *nuttallii* remains green during the winter and is able to recycle and withdraw nutrients from senescing plant parts for reuse [16]. Similarly, the combined effect of factors, *e*.*g*. season and resources, or calcium and phosphorus availability for plants, can be as important as each factor taken separately. In hardwater for example, phosphate availability is limited by apatite solubility [17], and can partly explain discrepancies between results of *in situ* experiments in waters of different mineral status [18, 4]. These findings gave clear evidence that phosphorus uptake performance of invasive macrophytes is dependent on many environmental and endogenous variables in interaction. However, the combined effects of these variables are poorly understood.

*E*. *nuttallii* succeeds as a competitive species in aquatic habitats and colonizes a wide trophic range of streams and ponds of the north eastern France [4, 19, 20]. This ability is due to its biological attributes, *e*.*g*. an efficient vegetative growth and a high phenotypic plasticity [21, 22] depending on the trophic state.

This work explores the mechanisms by which this invasive species adapts to its environment, by testing whether i) water phosphorus, ii) kind of water related to hardness and alkalinity (mineral status), iii) phenological phases and iv) subpopulation of *E*. *nuttallii* affect shoot phosphorus uptake kinetics and growth. Finally, this study provides data on phenotypic plasticity which is frequently suggested as an important mechanism of plant invasions but rarely investigated empirically ([23] and references therein). We thus carried out a microcosm-crossed experiment in order to assess the relative role and combined effects of these factors.

## Materials and Methods

### Materials

Native to North America, *E*. *nuttallii* was introduced into Europe in 1939 [24]. Only female plants were observed initially, introduced via the trade in live aquarium plants [25]. In the Rhine floodplain the species was first recorded in 1950 [26]. The occurrence of *E*. *nuttallii* in the Northern Vosges streams is the result of a single introduction event [22] and all the *Vosgian* subpopulations are clonal, whereas that of *E*. *nuttallii* in the *Alsatian* Rhine floodplain is probably the result of multiple events [27].

Field sampling did not involve endangered or protected species. The experiment was conducted both on *Vosgian* and *Alsatian* subpopulations. The *Vosgian* subpopulation was collected in the Moder tributary (49.00 N; 7.63 E). In this stream, *E*. *nuttallii* was first recorded during the 1970s [4]. The *Alsatian* subpopulation was collected in the drainage canal, an artificialized channel created along the Rhine River between 1930 and 1970 (48.29 N; 7.46 E).

### Experimental procedures

The two subpopulations of *E*. *nuttallii* were separately collected in December (winter experiment), May (spring experiment) and July (summer experiment) and placed in aquaria under experimental conditions: stream water (collected at the same time as the plants) with continuous bubbling for O_2_ to maintain 80–100% of oxygen saturation (Oxi 330i, WTW GmbH) and to supply CO_2_, 16°C±2°C, no sediment, 14:10 light:dark and under a light intensity of 5000 ± 200 lux. Experimental temperature and light were in the range of aquatic macrophyte active growth and is adapted to cultivation [28, 29]. The experimental temperature corresponds to the annual mean temperature of streams where plants have been harvested. After a four-day acclimatation period, 7 cm long shoots were cut and replaced in the aquaria, 24 hours before the beginning of the experiment. These selected parts of *E*. *nuttallii* are growing tips sensitive to phosphorus variations [30–33] and known to have the ability to absorb high quantities of phosphorus [15, 18, 34].

Two 72 hours experiments were consecutively carried out per season (winter, spring, summer, seasons for which P uptake and growth were supposed to change, according to field experiments [46]) in *Vosgian* softwater (pH = 7.2, conductivity = 72 μS.cm^−1^, mean values during the experiments) and in *Alsatian* hardwater (pH = 8.4, conductivity = 669 μS.cm^−1^, mean values during the experiments). According to the natural difference between mineral status (particularly water calcium content) of *Alsatian* Rhine floodplain hardwater streams compared to the Northern Vosges mountain softwater streams, both areas situated in north eastern France, we compared phosphorus uptake of *Vosgian* and *Alsatian E*. *nuttallii* subpopulations in their original water and by permuting waters. pH and conductivity were monitored daily with portable instruments (pH with pH 320, WTW GmbH, Weilheim, Germany, and conductivity with HI 98311, Hanna instruments, Woonsocket, RI, U.S.A.) and no significant variations during experiments were noticed.

At the beginning of each experiment, *Vosgian* and *Alsatian E*. *nuttallii* shoots were weighed and placed separately in aquaria. Each of the 12 aquaria (2 subpopulations * 2 P content * 3 repetitions) contained 6 l of stream water and 20 shoots. Five units of 20 supplementary shoots of both *E*. *nuttallii* subpopulations were used for determinations of initial dry mass and phosphorus content. Initial dry mass was (mean ± standard deviation) 0.38 g ± 0.02 for the winter experiment, 0.65 g ± 0.07 for the spring experiment, and 0.57 g ± 0.06 for the summer experiment. The experiment started by adding either 100 μg.l^−1^ P-PO_4_^3−^ (eutrophic conditions) or 300 μg.l^−1^ P-PO_4_^3−^ (hypertrophic conditions) to each aquarium. The whole experiment was repeated by permuting waters. During the summer experiment, aquaria without adding phosphorus were also performed (oligotrophic state), and showed an undetectable level of water phosphorus after an 18-hour experiment. Initial values of *Alsatian* P-PO_4_^3−^ water were < 5 μg.l^−1^ for spring, 11 μg.l^−1^ for winter and 10 μg.l^−1^ for summer. Initial values of *Vosgian* P-PO_4_^3−^ water were 28 μg.l^−1^ for spring, 34 μg.l^−1^ for winter and 37 μg.l^−1^ for summer.

In order to follow the disappearance of phosphorus, 50 ml of water was sampled in each aquarium after 0, 6, 12, 18, 24, 30, 36, 42, 48, 54, 60, 66 and 72 h of incubation under experimental conditions (see above). Phosphate disappearance was attributed to its absorption by plant (i.e. uptake) because preliminary experiments with antibiotic (chloramphenicol, unpublished data) showed that phosphorus absorption by microflora was not significant at concentrations lower than 400 μg.l^−1^ P-PO_4_^3−^, and rinsing and wiping up the shoots reduced strongly adhering organisms (unpublished data).

### Analytical methods

Calcium water content was analyzed by atomic absorption spectrophotometer (after adding a 0.2% solution of lanthane in the samples). Measurements concerned only the spring experiment. We analyzed water calcium content dynamics (i.e. at each sampling date) in one aquarium with *Vosgian E*. *nuttallii* subpopulation and one aquarium with *Alsatian E*. *nuttallii* subpopulation in the hypertrophic conditions and in both waters. Measurements were also performed in all the aquaria (corresponded to all treatments) at the initial sampling date, after 36 and after 72 hours of incubation.

At the end of the experiment and after the last sampling time (72 hours), we determined the final dry mass (60°C during 3 days) and total phosphorus content of the 20 *E*. *nuttallii* shoots pooled for each aquarium. Dried plants were crushed in a MM 2000 Retsch ball crusher and passed through a 2-mm sieve. Phosphate of water and plants (after nitro-perchloric mineralization of 5 g DM, [35]) was analyzed by colorimetry after chemical reaction (ammonium molybdate/ascorbic acid), with a microflow auto-analyzer (Alliance Instruments Integral ETC), according to the spectrometric method NF T 90-023.

### Data analysis

The experimental design was a complete randomized block, and no block’s effect was observed for all measured parameters (phosphorus and calcium water content, pH, conductivity, O_2_, plant P and growth; ANOVA, p > 0.05).

The phosphorus absorption was analyzed for each parameter (season, trophic state and water-subpopulation treatments) using simple linear regressions describing the decrease in phosphorus concentration (y, in μg.l^−1^ initial dry mass) as a function of time (x, in hours) [36]. The goodness of fit was tested by linearity test. Differences in the rate of phosphorus uptake were demonstrated using an analysis of covariance followed by a multiple comparison among slopes [36].

To assess the role of season, trophic state, water-subpopulation treatment and their combined effect on phosphorus uptake by plants, a four-way (season, trophic state, water and subpopulation) analysis of variance (ANOVA) was performed and followed by Tukey’s test for post-hoc pairwise comparisons, if overall differences were significant. If any interaction occurred between one studied factor with another, we excluded it before performing another ANOVA. Mean plant phosphorus concentrations were compared from the beginning to the end of the experiments (72h), or between conditions (season, trophic states and mineral levels of the water, *E*. *nuttallii* subpopulation) by performing Mann-Whitney tests, as normality and/or homoscedasticity were not observed.

In order to evaluate if the effect of environmental conditions (mineral and trophic states) on the functional trait “water phosphorus uptake” is linked to an increase of the performance trait (“growth rate”), we calculated phenotypic plasticity indexes for both *E*. *nuttallii* subpopulations in the different situations. Each index value represents (phosphorus uptake or growth rate in natural conditions, e.g. *Alsatian E*. *nuttallii* in *Alsatian* water or *Vosgian E*. *nuttallii* in *Vosgian* water) – (phosphorus uptake or growth rate when we changed water, e.g. *Vosgian E*. *nuttallii* in *Alsatian* water or *Alsatian E*. *nuttallii* in *Vosgian* water)/maximum uptake or growth rate.

Normality and homoscedasticity were tested before analyses, with Shapiro-Wilk and Levene tests, respectively.

Statistical analyses were conducted with Minitab© (release 13 for Windows 2000, Minitab SARL, Paris, France) and Statgraphics plus (version 4.1: Statistical Graphics Corporation©). Significant level was considered to be 5%.

## Results

### Water calcium concentrations

**Fig 1:**
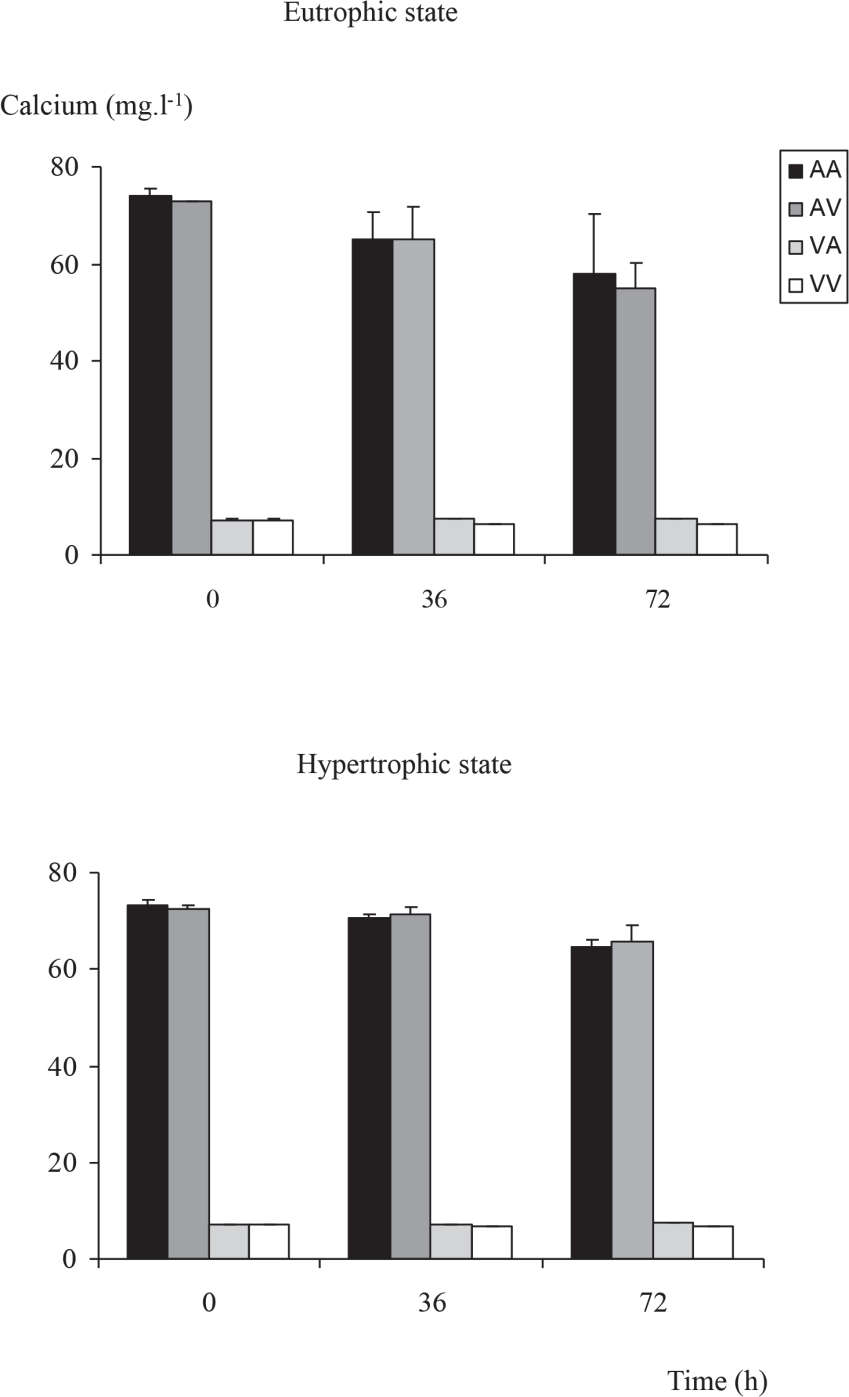
Dynamics of water calcium. Dynamics of calcium (in mg.l^−1^) contained in *Alsatian* or *Vosgian* water for eutrophic and hypertrophic conditions and for all the possible combinations of water and subpopulation. In the legend, AA, AV, VA, VV include the information on the treatment, the first letter refers to the type of water and the second one to the subpopulation (e.g. AV means *Alsatian* water and *Vosgian* subpopulation).

*Alsatian* water contained 10 times more calcium (between 70 and 80 mg.l^−1^) than *Vosgian* water (about 7 mg.l^−1^) (Fig. 1, three-way ANOVA, p < 0.001). Calcium decreased significantly from the beginning of the incubation to 36 or 72 h incubation (three-way ANOVA, p < 0.001) for both trophic states in hard and softwaters. More calcium was observed in hypertrophic conditions compared to eutrophic ones in *Alsatian* waters (three-way ANOVA, p < 0.01).

### Phosphorus uptake kinetics

**Fig 2:**
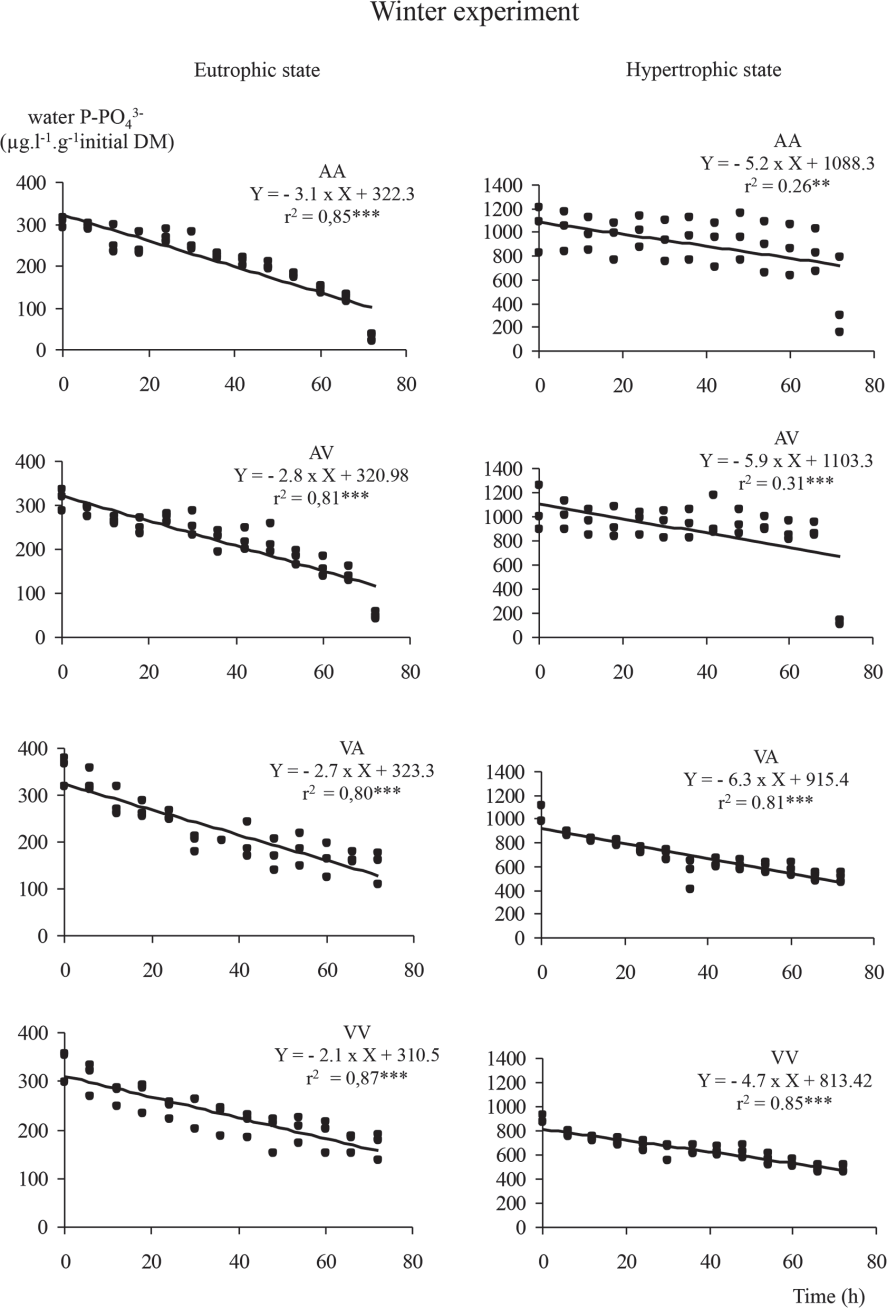
Dynamics of water phosphorus in winter. Dynamics of water phosphorus (in μg.l^−1^.g^−1^ of plant initial dry mass) and results of a linear model regression analyses under eutrophic and hypertrophic conditions, for all the possible combinations of water and subpopulation, during the winter experiment. In the legend, AA, AV, VA, VV include the information on the treatment, the first letter refers to the type of water and the second one to the subpopulation (e.g. AV means *Alsatian* water and *Vosgian* subpopulation).

**Fig 3:**
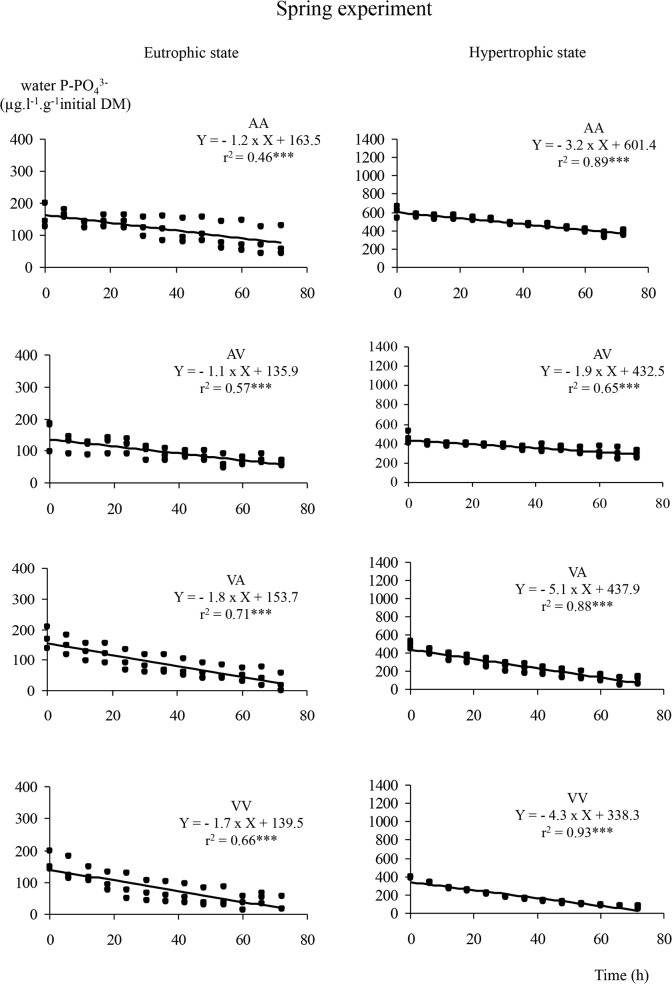
Dynamics of water phosphorus in spring. Dynamics of water phosphorus (in μg.l^−1^.g^−1^ of plant initial dry mass) and results of a linear model regression analyses under eutrophic and hypertrophic conditions, for all the possible combinations of water and subpopulation, during the spring experiment. In the legend, AA, AV, VA, VV include the information on the treatment, the first letter refers to the type of water and the second one to the subpopulation (e.g. AV means *Alsatian* water and *Vosgian* subpopulation).

**Fig 4:**
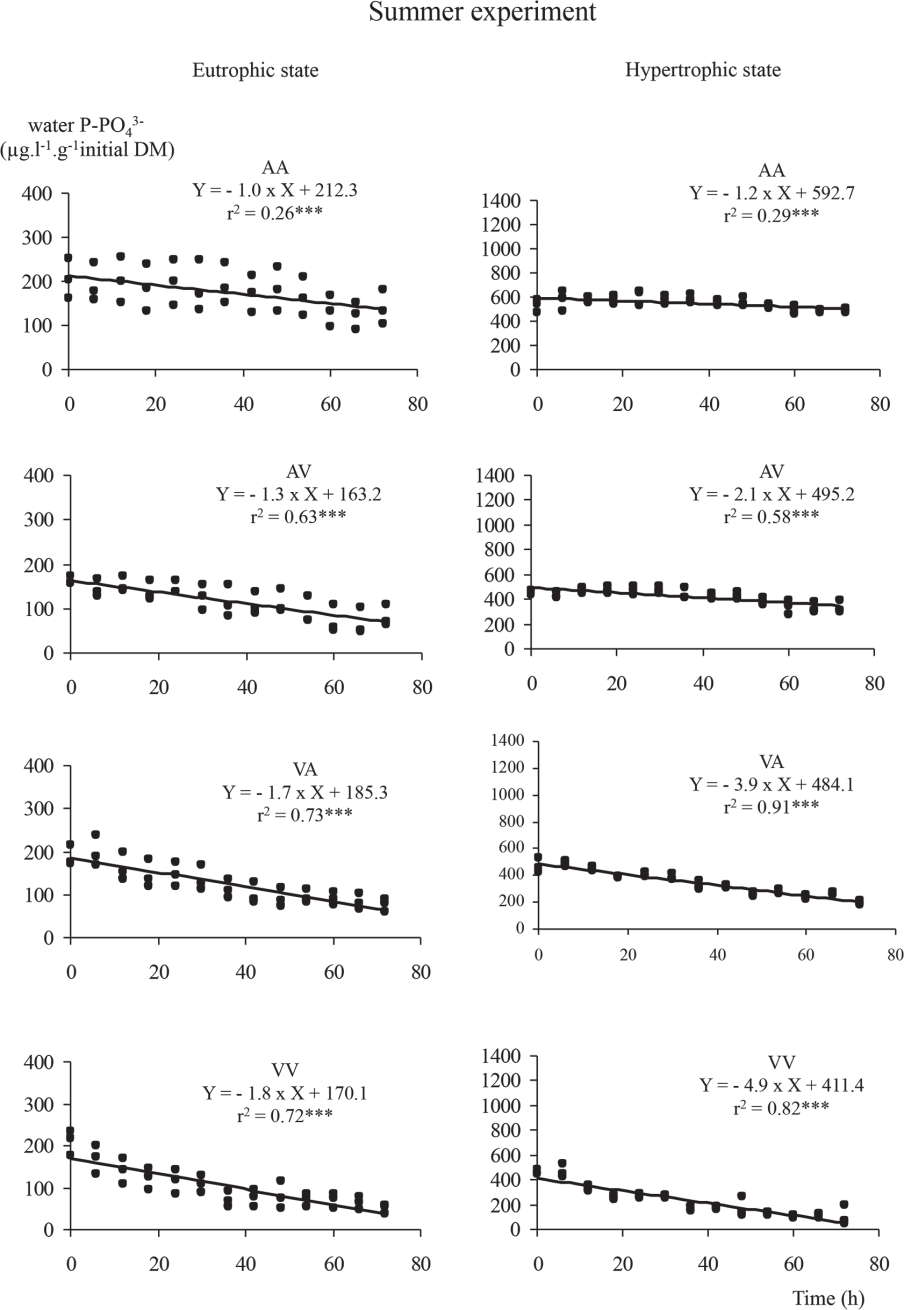
Dynamics of water phosphorus in summer. Dynamics of water phosphorus (in μg.l^−1^.g^−1^ of plant initial dry mass) and results of a linear model regression analyses under eutrophic and hypertrophic conditions, for all the possible combinations of water and subpopulation, during the summer experiment. In the legend, AA, AV, VA, VV include the information on the treatment, the first letter refers to the type of water and the second one to the subpopulation (e.g. AV means *Alsatian* water and *Vosgian* subpopulation).

Phosphorus dynamics showed a decrease of concentrations over time (Figs. 2, 3, 4) following a linear model (linearity test, p < 0.005) whatever *E*. *nuttallii* subpopulation, initial phosphorus or calcium water contents. Multiple comparisons of slopes, based on subpopulation and type of water (Table 1), showed that under eutrophic conditions and during winter experiment, the *Vosgian E*. *nuttallii* subpopulation in *Vosgian* water (VV) was less efficient in P uptake than the *Alsatian E*. *nuttallii* subpopulation in *Alsatian* water (AA) (covariance analysis, p < 0.05), but there was no difference between both subpopulations when permuting water (AV and VA). On the opposite, during spring experiment and in eutrophic conditions, a significant difference between treatments was observed only when *E*. *nuttallii* plants were grown in water with the reciprocal mineralization (AV and VA) (covariance analysis, p < 0.05). Thus, the *Alsatian E*. *nuttallii* subpopulation in *Vosgian* water (VA) absorbed phosphorus more efficiently than the *Vosgian E*. *nuttallii* subpopulation in *Alsatian* water (AV). During summer experiment and in eutrophic conditions, only the *Vosgian E*. *nuttallii* subpopulation in *Vosgian* water (VV) was more efficient than the *Vosgian E*. *nuttallii* subpopulation in *Alsatian* water (AV) and the *Alsatian E*. *nuttallii* subpopulation in both waters (AA and VA).

**Table 1:** Results of covariance analyses on the decrease of water phosphorus concentrations based on the rearing treatment (subpopulation and water).

Seasonal and trophic state parameters	F	p	Multiple comparisons among slopes post hoc tests
Winter—Eutrophic	3.76	< 0.05	AA^b^ AV^ab^ VA^ab^ VV^a^
Winter-Hypertrophic	0.50	ns	-
Spring-Eutrophic	3.38	< 0.05	AA^ab^ AV^a^ VA^b^ VV^ab^
Spring—Hypertrophic	26.22	< 0.001	AA^b^ AV^a^ VA^c^ VV^c^
Summer-Eutrophic	3.02	< 0.05	AA^a^ AV^a^ VA^a^ VV^b^
Summer-Hypertrophic	31.20	< 0.001	AA^a^ AV^a^ VA^b^ VV^b^

For each treatment, n = 39. The first letter of the acronym corresponds to the water (A for *Alsatian* and V for *Vosgian* water), the second letter corresponds to the subpopulation (A for *Alsatian* and V for *Vosgian* subpopulation) e.g. AV: *Vosgian E*. *nuttallii* in *Alsatian* water. The results of the multiple comparisons among slopes are given by an exponent letter: values that do not differ at the 0.05 level are noted with a same letter (a < b < c).

In hypertrophic conditions and during spring experiment, there was no difference in rate of phosphorus uptake for both subpopulations when they were placed in *Vosgian* water (VA and VV). In *Alsatian* water, the *Alsatian E*. *nuttallii* subpopulation (AA) showed higher phosphorus uptake than the *Vosgian* one (AV). In hypertrophic conditions and during summer experiment, the rate of phosphorus uptake was higher in *Vosgian* water than in *Alsatian* water. Consequently, both subpopulations of *E*. *nuttallii* absorbed phosphorus more efficiently when placed in *Vosgian* water (VV and VA).

Multiple comparisons among slopes based on season (Table 2) showed that uptake of phosphorus was the highest during winter whatever the water or subpopulation considered, except for *Alsatian E*. *nuttallii* in *Vosgian* water (VA) for which the uptake was similar between spring and winter and in *Vosgian* water (VV) for which the effect of season on rate of phosphorus uptake was not significant.

**Table 2:** Results of covariance analyses on the decrease of water phosphorus concentrations based on the season.

Treatment and trophic state parameters	F	p	Multiple comparisons among slopes post hoc tests
AA—Eutrophic	23.28	< 0.001	Winter^b^ Spring^a^ Summer^a^
AA—Hypertrophic	5.14	< 0.01	Winter^b^ Spring^a^ Summer^a^
AV—Eutrophic	26.70	< 0.001	Winter^b^ Spring^a^ Summer^a^
AV—Hypertrophic	6.77	< 0.01	Winter^b^ Spring^a^ Summer^a^
VA—Eutrophic	5.75	< 0.01	Winter^b^ Spring^a^ Summer^a^
VA—Hypertrophic	9.52	< 0.001	Winter^b^ Spring^b^ Summer^a^
VV—Eutrophic	1.44	ns	-
VV—Hypertrophic	0.97	ns	-

For each season, n = 39. The first letter of the acronym corresponds to the water (A for *Alsatian* and V for *Vosgian* water), the second letter corresponds to the subpopulation (A for *Alsatian* and V for *Vosgian* subpopulation) e.g. AV: *Vosgian E*. *nuttallii* in *Alsatian* water. The results of the multiple comparisons among slopes are given by an exponent letter: values that do not differ at the 0.05 level are noted with a same letter (a < b < c).

Multiple comparisons of slopes, based on trophic state (Table 3), showed that the rate of phosphorus uptake was higher in hypertrophic conditions compared to eutrophic ones, except during winter and summer experiments for *Alsatian E*. *nuttallii* in *Alsatian* water (AA) for which no significant effect of trophic state on rate of phosphorus uptake was observed.

**Table 3:** Results of covariance analyses on the decrease of water phosphorus concentrations based on the trophic state.

Treatment and season parameters	F	p	Multiple comparisons among slopes post hoc tests
AA—Winter	0.17	ns	-
AA—Spring	50.60	< 0.001	Eutrophic^a^ Hypertrophic ^b^
AA—Summer	0.63	ns	-
AV—Winter	4.40	< 0.001	Eutrophic^a^ Hypertrophic ^b^
AV—Spring	9.42	< 0.001	Eutrophic^a^ Hypertrophic ^b^
AV—Summer	5.20	< 0.05	Eutrophic^a^ Hypertrophic ^b^
VA—Winter	35.98	< 0.001	Eutrophic^a^ Hypertrophic ^b^
VA—Spring	73.08	< 0.001	Eutrophic^a^ Hypertrophic ^b^
VA—Summer	71.57	< 0.001	Eutrophic^a^ Hypertrophic ^b^
VV—Winter	45.54	< 0.001	Eutrophic^a^ Hypertrophic ^b^
VV—Spring	51.31	< 0.001	Eutrophic^a^ Hypertrophic ^b^
VV—Summer	53.96	< 0.001	Eutrophic^a^ Hypertrophic ^b^

For each trophic state, n = 39. The first letter of the acronym corresponds to the water (A for *Alsatian* and V for *Vosgian* water), the second letter corresponds to the subpopulation (A for *Alsatian* and V for *Vosgian* subpopulation) e.g. AV: *Vosgian E*. *nuttallii* in *Alsatian* water. The results of the multiple comparisons among slopes are given by an exponent letter: values that do not differ at the 0.05 level are noted with a same letter (a < b < c).

### Phosphorus content and plant growth

Mean plant phosphorus concentration was 0.61% ± 0.15 of plant dry mass (ranging from 0.3–1% of plant dry mass) and did not significantly differ from the beginning to the end of the experiments (72h), or between conditions (season, trophic states and mineral levels of the water, *E*. *nuttallii* subpopulation) (Mann-Whitney, p > 0.05).

**Fig 5:**
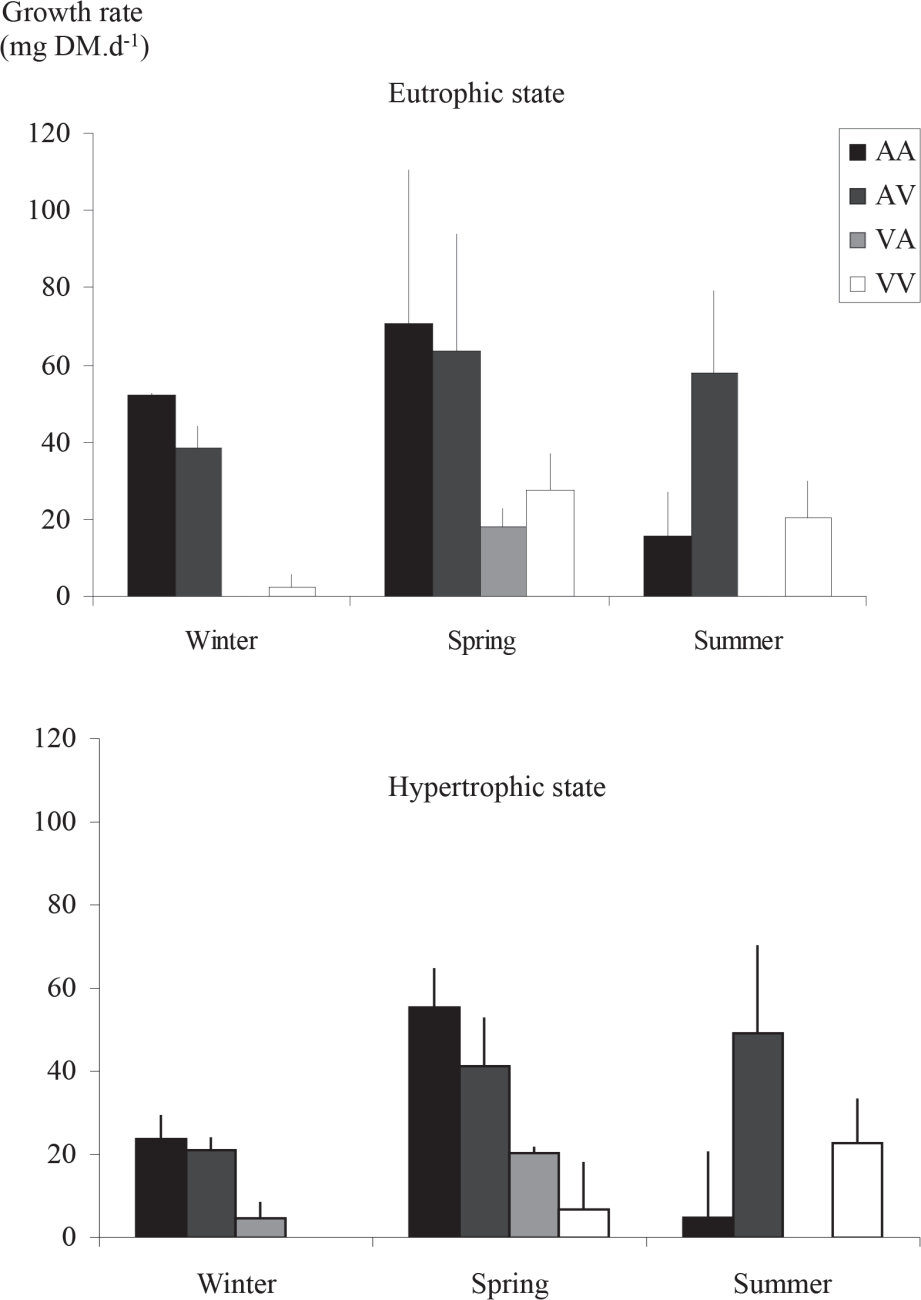
Plant growth rate. Plant growth rate (in mg of plant dry mass per day) under eutrophic and hypertrophic conditions, for all the possible combinations of water and subpopulation and during the winter, spring and summer experiments. In the legend, AA, AV, VA, VV include the information on the treatment, the first letter refers to the type of water and the second one to the subpopulation (e.g. AV means *Alsatian* water and *Vosgian* subpopulation).

Growth rate of *E*. *nuttallii* varied according to season (four-way ANOVA, p < 0.001; Tukey test, spring > summer > winter, p < 0.001), water (four-way ANOVA, p < 0.001; Tukey test, *Alsatian* > *Vosgian*, p < 0.0001), subpopulation (four-way ANOVA, p < 0.05; Tukey test, *Vosgian* > *Alsatian*, p < 0.05), and trophic state (four-way ANOVA, p < 0.01; Tukey test, eutrophic > hypertrophic, p < 0.01) (Fig. 5). However, interactions between season and subpopulation (four-way ANOVA, p < 0.001) and the type of water and trophic state (four-way ANOVA, p < 0.05) were also significant.

During summer experiment, the *Vosgian E*. *nuttallii* subpopulation showed a higher growth rate than the *Alsatian E*. *nuttallii* subpopulation (three-way ANOVA, p < 0.001, and Tukey test, p < 0.0001) whereas during winter experiment the opposite was observed (three-way ANOVA and Tukey test, p < 0.05). Finally, during spring experiment, for which the growth rate was highest, no difference between the two subpopulations was determined (three-way ANOVA, p > 0.05).

Compared to hypertrophic conditions, eutrophic state favoured the growth of *E*. *nuttallii* in *Alsatian* water (three-way ANOVA, p < 0.05, and Tukey test, p < 0.01), whereas trophic state did not have any effect on plant growth in *Vosgian* water (three-way ANOVA, p > 0.05).

### Phenotypic plasticity indexes

Results of phenotypic plasticity indexes showed that the functional trait “phosphorus uptake” was impacted by environmental conditions (mineral and trophic states) for both subpopulations, and this effect is linked with a change in performance trait (growth rate) (Table 4). During winter, phenotypic plasticity allowed *Alsatian* subpopulations to benefit from a changing environment (*i*.*e*. mineral water status) by increasing its performance whatever the trophic state. However, *Vosgian* subpopulation suffered a passive change in environmental conditions, as phenotypic plasticity indexes were negative both for P uptake and growth rate, regardless the trophic state. On the opposite, during spring, change of water led to a decrease of P uptake for *Alsatian* subpopulation and an increase for *Vosgian* one. However, the increase of P uptake for *Vosgian* subpopulation led to a decrease of plant growth rate, indicating an important cost for the plant to adapt to this new environment and/or a P storage behaviour. During summer, reactions were mixed between winter and spring, with a strong effect of trophic state, as permutation of water was negative on P uptake under eutrophic conditions but positive under hypertrophic conditions. This result was observed for both subpopulations but particularly for *E*. *nuttallii* coming from *Alsatian* floodplain.

**Table 4:** Phenotypic plasticity indexes for both subpopulations calculated from phosphorus uptake (functional trait) and growth rate (performance trait) data.

Population, trophic state and season parameters	P uptake	Growth rate
A—Eutrophic—Winter	0.39	1
V—Eutrophic—Winter	−0.44	−0.94
A—Hypertrophic—Winter	0.26	0.81
V—Hypertrophic—Winter	−0.54	−1
A—Eutrophic—Spring	−0.33	0.74
V—Eutrophic—Spring	0.25	−0.57
A—Hypertrophic—Spring	−0.33	0.63
V—Hypertrophic—Spring	0.44	−0.84
A—Eutrophic—Summer	−0.31	1
V—Eutrophic—Summer	−0.85	−0.65
A—Hypertrophic—Summer	0.73	1
V—Hypertrophic—Summer	0.37	−0.54

Each index value represents (phosphorus uptake or growth rate in natural conditions, e.g. AA or VV treatments)–(phosphorus uptake or growth rate when we changed water, e.g. VA or AV treatments)/maximum uptake or growth rate.

## Discussion

### Performance of *E*. *nuttallii* in water phosphorus uptake

Plant phosphorus concentrations were above 0.3%, which is much higher than the critical value for maximum yield (0.13% of leaf dry mass) of aquatic vascular plants mentioned by Gerloff & Krombholz [30] but in the range of previous studies for the same species [2, 18, 37]. Therefore, in microcosm conditions and without sediment, *E*. *nuttallii* was able to uptake phosphorus from water by shoots, covering its growth needs and even exhibiting “luxury” consumption [2, 3, 37]. In addition, no threshold was noticed after three days of P uptake, thus indicating that an increase in P availability can lead to an increase in P uptake [4, 38]. This ability to absorb nutrients with shoots when P-levels are at very high rates represents the features of competitive strategy of the species [8]. As a consequence, nutrient levels are reduced in the water column and can limit growth of other plants, especially free-floating plants [39].

However, plant phosphorus content did not increase significantly during the experiment, whereas the values were in the range of previous studies for the same species [2, 18, 37]. Nutrient-use efficiency is a function of nutrient availability, biomass allocation and growth rate [37, 40]. A large supply of nutrients may boost growth and, therefore, lead to relatively lower biomass produced per milligram of elements absorbed (dilution effect) [40]. As we observed an increase of mass from the beginning to the end of the 3-day fertilization experiment, this observation is fully tenable and confirmed that *E*. *nuttallii* has a high capacity for mobilising phosphorus accumulated in its tissue for growth [2].

Plant growth rate was lower (*Alsatian* water) or similar (*Vosgian* water) under hypertrophic compared to eutrophic conditions, indicating that the efficiency with which plants used nutrients declined with increased supply of P [8, 37]. Moreover, in several cases, luxury consumption could occur as indicated by the greatly enhanced tissue nutrient concentrations after fertilization [37].

### Importance of season, water mineral and trophic states, and subpopulation in phosphorus uptake by *E*. *nuttallii*

Water trophic state seems to be the main factor governing P uptake by these aquatic macrophytes. Mineral status of the stream water and to a lesser extent the season, also affected the performance of *E*. *nuttalli* in water P uptake.

High water trophic state favoured P uptake by *E*. *nuttallii* species, as P uptake rates were generally significantly higher in hypertrophic than in eutrophic conditions. Likewise, several authors clearly described the dependency of phosphorus uptake efficiency on the surrounding water phosphorus content [4, 18, 38].

Although the extrapolation of mesocosms experiment results to natural systems is inherently difficult [41], we could generalize the capacity of this invasive species to increase P uptake in response to water P availability, regardless of water mineralization, season or plant population.

Regarding mineral status (hardness and alkalinity), the present study showed that phosphorus uptake was generally higher in *Vosgian* softwater than in *Alsatian* hardwater, and that this effect is stronger in hypertrophic waters. As we observed a significant decrease of calcium during the course of the experiments, we can hypothesize that orthophosphates are likely combined with calcium to form apatite (Ca5(PO_4_)3OH) in alkaline waters [17], a form of phosphorus which is unavailable to macrophytes [42]. The change of water seemed to be favourable to a biomass increase, which can be explained by the presence of nutrient limiting conditions, as for calcium or carbonate. Calcium might also have a synergistic effect on growth of *Vosgian E*. *nuttallii* subpopulation, by using CaCO_3_ as an additional carbon source for photosynthesis [43], especially compared to softwater where bicarbonate can constitute a limiting factor [44].

Plant responses also differed as a function of season. The maximum P uptake was observed during winter, whereas spring and summer uptakes were similar. This result is consistent with observations performed in natural conditions [3]. However, the seasonal variability in P uptake is a confounding factor with water trophic states, as winter is the season with the lowest water P concentrations in natural conditions [31, 45]. Even though available P was not limiting as we added directly PO_4_^3−^ for these microcosm experiments, changes in P uptake with season cannot be explained only by phenology since *E*. *nuttallii* seems to be able to adapt their phosphorus uptake strategy to the availability of the different kinds of phosphorus sources [15]. Nonetheless, phenology must play a role in plant P-uptake. In winter, when the growth rate is the lowest although P-uptake is the highest, the nutrient-use efficiency is the lowest. This reveals a special fate of P storage, even if the increase of plant P was not significant after our experiments, which can be explained by the large amount of P already stored in plants. The new shoots developing at the end of autumn, described as one of the three growth stages in natural systems [22], probably served in phosphorus accumulation for the next year’s initial growth for *E*. *nuttallii*. Indeed, the highest growth rate was observed during spring, concurrent with a low nutrient uptake but the highest nutrient-use efficiency. The ability to remain in a vegetative condition throughout the winter season and rapid growth in spring is part of *Elodea* success as an invasive genus.

In this study, we showed that the high capacity of aquatic macrophytes to adjust their ecophysiology depends on both the environmental conditions and the plant geographical origins [46]. In Western Europe, as only female plants and clonal reproduction were observed, the wide range of morphological variations within *E*. *nuttallii* species is entirely due to phenotypic plasticity [21, 22, 25].

Phenotypic plasticity in functional traits as the phosphorus uptake, can allow invasive plants to benefit from changing environmental conditions [23]. However, plasticity of morphological and physiological traits is unlikely to have any effect on invasiveness unless that plasticity contributes to fitness [23]. Results of the present study confirmed that *E*. *nuttallii*, at least the *Alsatian* subpopulation, could strongly act as an invasive plant by showing positive plasticity for fitness.

We can assume that selection favors genotypes of introduced species with higher allocation towards competitive ability and growth [47]. The shift of allocation to growth in the introduced populations will enhance the settlement and proliferation of the exotic plant in the introduced range, which explains some differences in response between the two subpopulations of *E*. *nuttallii* tested.

## Conclusion

Our results indicate that P uptake was governed firstly by water trophic state and secondly by water mineral status. Several interactions between the tested factors, which prevented defining the relative importance of the other factors affecting P uptake such as phenology and subpopulations were determined. Finally, phenotypic plasticity allows both subpopulations of *E*. *nuttallii* to adapt to a changing environment.
